# Height at three months can indicate overweight at two years in catch-up growth of small for gestational age infants

**DOI:** 10.1038/s41598-018-29698-8

**Published:** 2018-09-07

**Authors:** Shi Chen, Zeyu Liu, Huijuan Zhu, Hongbo Yang, Fengying Gong, Linjie Wang, Yu Jiang, Chengsheng Yan, Jianqiang Li, Qing Wang, Hui Pan

**Affiliations:** 1 Department of Endocrinology, Key Lab of Endocrinology, National Commission of Health, Peking Union Medical College Hospital (PUMCH), Chinese Academe of Medical Sciences & Peking Union Medical College (CAMS & PUMC), Beijing, 100730 China; 2 Department of Radiology, PUMCH, CAMS & PUMC, Beijing, 100730 China; 3 School of Public Health, PUMC, Beijing, 100730 China; 4 Hebei Center for Women and Children’s Health, Shijiazhuang, 050031 China; 5 School of Software Engineering, Beijing University of Technology, Beijing, 100124 China; 6 Tsinghua National Laboratory for Info. Science and Technology, Tsinghua University, Beijing, 100084 China

## Abstract

This study aimed to find an indicator at three months to predict overweight and short stature at two years in small for gestational age (SGA) infants. A total of 468 SGA infants and 4642 appropriate for gestational age (AGA) infants were included. Weight and height were measured at birth, three months and two years. Logistic regression and receiver operating characteristic (ROC) curves were performed for the catch-up growth. As compared to AGA infants, the weight of SGA infants was lower and the length/height was shorter at birth, three months, and two years. The weight of the catch-up group was significantly greater at birth and two years. The length/height of the catch-up group was greater at three months and two years. Trajectories of weight standard deviation score (SDS) and height SDS showed that the overweight group (BMI over the 85^th^ percentile) had a shorter length/height SDS but a higher rate of the change in weight SDS during catch-up growth. The multivariate logistic regression indicated that that height at three months was an independent factor for prediction of catch-up growth at two years. The area under curve (AUC) was 0.801 with the 95% confidence interval (CI) from 0.726 to 0.876. Therefore, height at three months can predict overweight at two years.

## Introduction

Birth weight is an important indicator of neonatal health^1,2^. Low birth weight can be expressed as small for gestational age (SGA), which is generally defined as the birth weight under 2 standard deviation (SD) below the mean or less than the 3^rd^ or 10^th^ percentile for the gestational age^3^. Epidemiological studies have shown that SGA births are complicated by maternal, placental and fetal factors^4^. SGA infants have a higher incidence of short stature in adulthood^5^. About 14% of short stature in adulthood was due to SGA at birth^6^. Although approximately 70–90% of SGA infants experienced catch-up growth, which was defined as −2SD score (SDS) or 3^rd^ percentile in height, 10% of SGA infants presented with short stature in adulthood, defined as <−2SDS or the 3^rd^ percentile in height^6–10^.

Many studies suggested that catch-up growth was accompanied by overweight/obesity and other diseases in later life^11–15^. Laitinen *et al*. concluded that SGA was an independent risk factor for abdominal obesity with the odds ratio (OR) ranging from 1.41 to 2.09 in different models after adjustments^16^. Moreover, metabolic syndrome including type 2 diabetes mellitus and cardiovascular disease were reported to be associated with SGA subjects in adulthood^17–19^. Thus, SGA infants faced long-term disadvantages and were at high metabolic risk^3,20^. Despite these findings indicating a relationship between SGA and metabolic risks, the underlying mechanism remains largely unknown^21^.

In most cases, the catch-up growth occurred before the age of two years^6–8,22,23^. In addition, the weight gain within two years was regarded as a predictor for overweight at school entry^12^. Hence, the age of two years was an important time point for subsequent overweight prediction. Thus, this study was designed as a two-year observational study to examine the catch-up growth trajectory of SGA infants in order to find a possible indicator at three months for the prediction of overweight and short stature at two years. This could help to detect and prevent overweight in later life.

## Methods

### Study design

**Figure 1:**
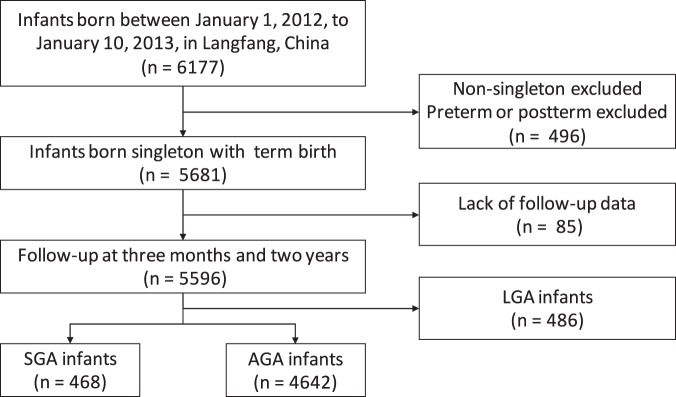
Flowchart of the study population. SGA, small for gestational age; AGA, appropriate for gestational age; LGA, large for gestational age.

The data used in this study were obtained from the database of the Observation Project on Growth of Children (OPGC), which was a population-based, cohort study conducted in Langfang, a city of five million people in north China. In order to increase the general quality of life of the population, OPGC was implemented by the Langfang maternal and child care service center. The project maintained medical records of the recruited couples and their children, which contained information of birth, including gestational age, gender, birth weight, birth length, placental weight, umbilical cord length, and the one-minute, two-minute and five-minute Apgar scores^24^. The basic information of mothers was also collected, including age, occupation, education, the history of childbirth, and blood pressure immediately after delivery and after two hours. All subjects were required to undergo regular physical examination at three months and two years of postnatal age. The information was recorded in a web-based electronic data collection system and sent to the national data center. A total of 6177 infants, born between January 1, 2012, and January 10, 2013, were included in this study. Gestational age was calculated from the first day of the last menstrual period to birth. There were 5681 infants after term birth (37–42 weeks of gestation age) and singleton selection. The definition of SGA was less than the 10^th^ percentile for the gestational age according to Chinese Neonatal Network^25^. Finally, 468 SGA subjects and 4642 appropriate for gestational age (AGA) subjects were analyzed. The flowchart of the study population is shown in Fig. 1. All parents signed the informed consent before participation. During the study design and manuscript preparation, the guideline from the STROBE statement was followed^26^.

### Data collection

The clinical data from physical examinations conducted by experienced medical staff, such as Apgar scores of each infant, were recorded. The weight and length/height was measured by trained nurses after removing shoes, bulky clothing and other factors that may influence the accuracy of measurement. Systolic blood pressure (SBP) and diastolic blood pressure (DBP) of mothers were measured using sphygmomanometer. Body mass index (BMI) was calculated as weight in kilograms divided by the square of height in meters. BMI over the 85^th^ percentile was defined as overweight in the current study^11^. SDS for weight and length/height were calculated based on gender and age. The calculation in this study was based on the Chinese standard^27,28^. Moreover, basic information including age, occupation, education, and the history of childbirth were noted from answers to a standardized questionnaire.

### Statistical analysis

Statistical analysis was performed using SPSS, version 16 (SPSS, Chicago, IL, USA). Continuous variables are presented as mean ± standard deviation with normal distribution or median (first quartile – third quartile) with skewed distribution. Quantitative variables were examined by the Student’s t-test for normal distribution or the Mann-Whitney U test for skewed distribution. In this study, subjects who achieved at least −2SDS in height at two years were defined as catch-up growth. Multivariate logistic regression was utilized to test the factors affecting the catch-up growth. Moreover, a receiver operating characteristic (ROC) curve was applied to test the sensitivity and specificity as well as the cut-off point for the diagnostic predictive model of catch-up growth. P < 0.05 was considered as statistical significance.

### Ethics statement

The study was approved by the ethics committee of the Peking Union Medical College Hospital in China and conducted in accordance with the Helsinki Declaration.

## Results

### Characteristics of the study population

As shown in Fig. 1, 468 infants were diagnosed as SGA and 4642 were AGA. The baseline characteristics of SGA and AGA subjects are displayed in Table 1. As compared to AGA subjects, the weight of SGA subjects was lower and the length/height was shorter. Also, SGA subjects had lower Apgar scores, lower placental weight and shorter umbilical cord length than AGA subjects.

**Table 1:** Characteristics of the study population.

Variable	SGA (n = 468)	AGA (n = 4642)	*P* value
Gender (M/F)	220/248	2507/2135	0.005*
Age of mothers (year)	24.78 ± 4.64	25.33 ± 4.33	<0.001*
Gravidity	1.53 ± 0.86	1.64 ± 0.87	0.001*
Parity	1.13 ± 0.74	1.23 ± 0.72	0.003*
Gestational age (week)	39.49 ± 0.98	39.28 ± 1.06	<0.001*
SBP-0 h (mmHg)	105.38 ± 21.32	110.83 ± 16.12	<0.001*
DBP-0 h (mmHg)	80.79 ± 12.56	76.60 ± 10.73	<0.001*
SBP-2 h (mmHg)	105.71 ± 18.46	110.55 ± 14.48	<0.001*
DBP-2 h (mmHg)	81.45 ± 14.63	76.92 ± 11.95	<0.001*
Placental weight (g)	473.66 ± 69.11	498.95 ± 76.69	<0.001*
Umbilical cord length (cm)	51.56 ± 5.95	52.11 ± 5.48	0.010*
One-minute Apgar scores	9.74 ± 0.64	9.78 ± 0.60	0.191
Two-minute Apgar scores	9.85 ± 0.46	9.94 ± 0.34	<0.001*
Five-minute Apgar scores	9.87 ± 0.39	9.94 ± 0.42	<0.001*
Birth weight (kg)	2.62 ± 0.24	3.36 ± 0.29	<0.001*
Weight at 3 months (kg)	6.39 ± 0.65	6.58 ± 0.68	<0.001*
Weight at 2 years (kg)	12.47 ± 0.77	12.61 ± 0.82	0.001*
Birth length (cm)	49.22 ± 1.89	50.12 ± 0.97	<0.001*
Height at 3 months (cm)	60.96 ± 1.88	61.60 ± 1.66	<0.001*
Height at 2 years (cm)	87.58 ± 1.93	87.90 ± 2.03	0.011*

Data are presented as mean ± standard deviation.

SGA: small for gestational age; AGA: appropriate for gestational age; SBP: systolic blood pressure; DBP: diastolic blood pressure; 0 h: immediately after delivery; 2 h: 2 hours after delivery.

*Significant differences with *P* value < 0.05.

### Trajectory of the SDS and catch-up overweight

**Figure 2:**
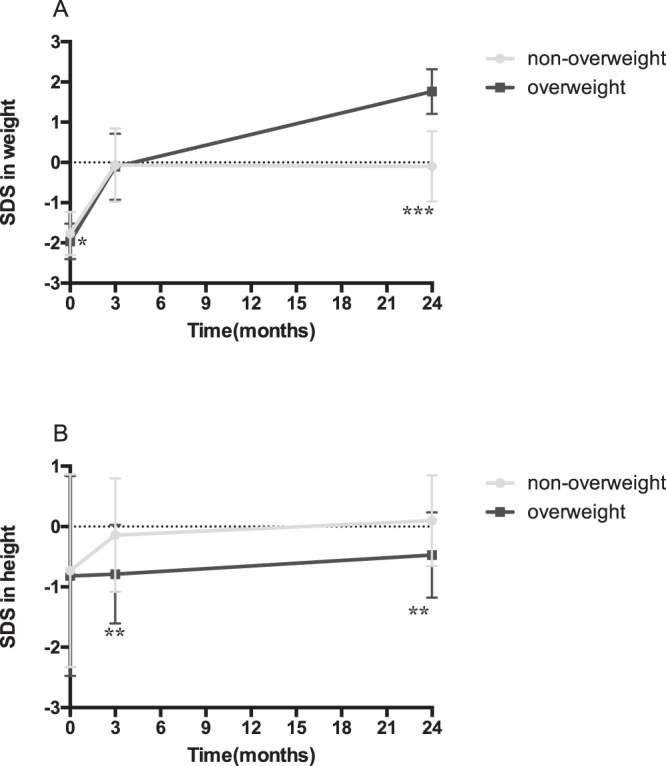
Trajectory of the SDS in weight (**A**) and height (**B**) in SGA infants in the first two years. *Significant differences with *p* < 0.05. **Significant differences with *p* < 0.01. ***Significant differences with *p* < 0.001.

A total of 432 out of 468 SGA subjects completed height catch-up at two years. However, 4.4% (19/432) of the subjects were overweight, which was defined as BMI over the 85^th^ percentile. Table 2 shows that birth weight and birth length were comparable between the groups. Gradually, subjects in the overweight group became shorter at three months, whereas the weight remained comparable between the groups. At the age of two years, subjects in the overweight group were shorter and heavier, with a larger BMI. Weight SDS and height SDS at birth, three months and two years are displayed in Fig. 2 to show the trajectories of the overweight and non-overweight groups. The weight SDS in the overweight group was significantly lower at birth, and the difference between the groups disappeared at three months. However, the weight SDS in the overweight group was significantly higher than that of the non-overweight group at two years. Figure 2A shows that the line representing the overweight group had a sharper slope in the change of weight SDS from three months to two years. No difference in height SDS was found at birth between the groups. A major difference in height SDS occurred at three months, indicating that the rate of catch-up growth in height was significantly different in the first three months. After three months, two lines in Fig. 2B are almost parallel to each other, suggesting that subjects in the overweight group were continually shorter than subjects in the non-overweight group. Therefore, the overweight group had a larger SDS in weight and a smaller SDS in height at the age of two years, accounting for the larger BMI.

**Table 2:** Overweight during catch-up growth in small for gestational age subjects.

Variable	Non-overweight (n = 413)	Overweight (n = 19)	*P* value
Gender (M/F)	194/219	11/8	0.352
Age of mothers (year)	24.73 ± 4.55	25.32 ± 6.05	0.709
Gravidity	1.53 ± 0.89	1.50 ± 0.67	0.790
Parity	1.11 ± 0.76	1.25 ± 0.75	0.408
Gestational age (week)	39.55 ± 0.97	39.37 ± 0.83	0.273
SBP-0 h (mmHg)	105.23 ± 21.49	100.21 ± 24.38	0.638
DBP-0 h (mmHg)	80.88 ± 12.61	86.95 ± 10.92	0.021*
SBP-2 h (mmHg)	105.53 ± 18.63	100.74 ± 19.20	0.269
DBP-2 h (mmHg)	81.66 ± 14.60	85.63 ± 16.39	0.189
Placental weight (g)	473.77 ± 70.98	475.26 ± 53.37	0.764
Umbilical cord length (cm)	51.64 ± 6.03	51.79 ± 5.07	0.923
One-minute Apgar scores	9.75 ± 0.60	9.89 ± 0.32	0.300
Two-minute Apgar scores	9.85 ± 0.46	9.89 ± 0.32	0.783
Five-minute Apgar scores	9.88 ± 0.39	9.89 ± 0.32	0.954
Birth weight (kg)	2.63 ± 0.23	2.56 ± 0.19	0.073
Weight at 3 months (kg)	6.41 ± 0.62	6.42 ± 0.61	0.851
Weight at 2 years (kg)	12.50 ± 0.63	13.84 ± 0.44	<0.001*
Birth length (cm)	49.29 ± 1.79	49.21 ± 1.87	0.835
Height at 3 months (cm)	61.14 ± 1.70	60.11 ± 1.56	0.005*
Height at 2 years (cm)	88.01 ± 1.36	87.11 ± 1.27	0.009*

Data are presented as mean ± standard deviation.

SBP: systolic blood pressure; DBP: diastolic blood pressure; 0 h: immediately after delivery; 2 h: 2 hours after delivery.

*Significant differences with *P* value < 0.05.

### Catch-up growth in SGA subjects

At the age of two years, 92.3% (432/468) of the SGA subjects achieved at least −2SDS in height, and were allocated to the catch-up group. As shown in Table 3, the catch-up group had larger gestational age than the non-catch-up group. The average birth weight of the non-catch-up group was significantly lower, but their birth length was equivalent. Gradually, the length between the groups changed whereas the weight was comparable at three months. At the age of two years, subjects in the catch-up group were 5 cm taller than the non-catch-up group. Accordingly, subjects in the catch-up group had a higher body weight at two years, with comparable BMI between the groups (16.20 vs. 16.25 kg/m^2^, *p* = 0.496).

**Table 3:** Catch-up growth in small for gestational age subjects.

Variable	NCU (n = 36)	CU (n = 432)	*P* value
Gender (M/F)	15/21	203/229	0.504
Age of mothers (year)	25.06 ± 5.00	24.76 ± 4.62	0.949
Gravidity	1.52 ± 0.68	1.53 ± 0.88	0.635
Parity	1.23 ± 0.62	1.12 ± 0.76	0.302
Gestational age (week)	38.86 ± 0.99	39.54 ± 0.97	<0.001*
SBP-0 h (mmHg)	109.86 ± 16.91	105.01 ± 21.62	0.717
DBP-0 h (mmHg)	76.58 ± 11.53	81.15 ± 12.59	0.020*
SBP-2 h (mmHg)	110.44 ± 15.25	105.32 ± 18.66	0.238
DBP-2 h (mmHg)	76.86 ± 13.25	81.84 ± 14.68	0.049*
Placental weight (g)	471.67 ± 54.17	473.83 ± 70.26	0.269
Umbilical cord length (cm)	50.53 ± 5.57	51.65 ± 5.98	0.789
One-minute Apgar scores	9.61 ± 1.08	9.75 ± 0.59	0.640
Two-minute Apgar scores	9.81 ± 0.47	9.85 ± 0.46	0.451
Five-minute Apgar scores	9.81 ± 0.47	9.88 ± 0.39	0.276
Birth weight (kg)	2.45 ± 0.27	2.63 ± 0.23	<0.001*
Weight at 3 months (kg)	6.17 ± 0.91	6.41 ± 0.62	0.073
Weight at 2 years (kg)	11.37 ± 0.96	12.56 ± 0.68	<0.001*
Birth length (cm)	48.42 ± 2.68	49.28 ± 1.80	0.058
Height at 3 months (cm)	59.31 ± 2.84	61.10 ± 1.71	<0.001*
Height at 2 years (cm)	82.88 ± 1.39	87.97 ± 1.36	<0.001*

Data are presented as mean ± standard deviation.

CU: catch-up growth; NCU: non-catch-up growth; SBP: systolic blood pressure; DBP: diastolic blood pressure; 0 h: immediately after delivery; 2 h: 2 hours after delivery.

*Significant differences with *P* value < 0.05.

The multivariate logistic regression was utilized with forward selection to investigate the predictors for catch-up prediction at two years with the independent variable from Table 3. Factors, such as gender, weight and Apgar scores were included in the multivariate logistic regression analysis, and the results before and after adjustments are shown in Table 4. The *p* values were always <0.001, which indicated that height at three months was an independent factor for prediction of catch-up growth at two years. The result was presented as logit (P) = 0.483 *height at 3 months + 0.422 *gestational age – 43.281. ROC curve was also established. The area under curve (AUC) was 0.801 with the 95% confidence interval (CI) from 0.726 to 0.876.

**Table 4:** Independent predictors for catch-up growth at two years according to height at three months in unadjusted and adjusted models.

Model	OR (95% CI)	*P* value
unadjusted	1.503 (1.261–1.790)	<0.001
adjusted for gender and weight at three months	1.627 (1.315–2.014)	<0.001
adjusted for gender, weight at three months, birth length, birth weight and one-, two-, five-minute Apgar scores	1.627 (1.298–2.040)	<0.001
adjusted for gender, weight at three months, birth length, birth weight, one-, two-, five-minute Apgar scores, placental weight, umbilical cord length and gestational age	1.621 (1.284–2.045)	<0.001

Odds ratios were determined using logistic regression analyses.

OR: Odds ratios; CI: confidence intervals.

## Discussion

In the current study, we investigated the trajectories of postnatal growth of SGA subjects from birth to the age of two years in order to detect early indicators for overweight of SGA subjects during catch-up growth and prevent overweight/obesity in later life. The results suggested that anthropometric features at three months were strongly related to those at the age of two years, which may be utilized as a predictor for overweight and short stature in later life.

The prevalence of SGA subjects with term birth who achieved catch-up was 92.3% (432/468) and 90.6% (424/468) in height and weight, respectively, which was almost the same as the Japanese cohort^7^. Moreover, Itabashi *et al*. reported that gestational age affected catch-up rate^7^. Similarly, Maeyama *et al*. found that height and BMI trajectories in the first three years were dependent on gestational age^20^. Although the underlying mechanism of how gestational age affects the catch-up growth is not fully understood, higher gestational age may facilitate catch-up growth. Accordingly, we found that subjects in the catch-up group had higher gestational age than those in the non-catch-up group. In the current study, the placental weight of SGA subjects was lower than AGA subjects, which was in accordance with a previous study that showed that lighter placenta provided insufficient substance^29^.

The trajectories of postnatal growth of SGA subjects were previously explored^7,30^. Crume *et al*. investigated the long-term impact of infants exposed to intrauterine growth restriction (IUGR), most of whom were SGA^30^. They showed that the 42 subjects exposed to IUGR experienced catch-up growth with a BMI growth rate of 3.58 kg/m^2^, which was greater than that of 464 unexposed subjects (2.36 kg/m^2^) in the first year. However, there were no differences in BMI growth trajectory after the first year^30^. Moreover, early signs of insulin resistance were detected in subjects exposed to IUGR, which prompted us to explore ways to minimize or avoid overweight/obesity during catch-up growth. Many researchers investigated the predictors for catch-up growth or overweight/obesity in adulthood^8,12^. Birth weight SDS was regarded as the best predictor for catch-up to the 3^rd^ percentile in SGA infants with term birth^8^. Our study showed that height at three months was better than birth weight SDS for catch-up prediction. As for overweight during catch-up growth, weight gain from birth to two years could predict subsequent overweight, for which the AUC was 0.76^12^.

As compared to the above-mentioned studies, the most striking result in this study was an earlier inflection point for the prevention of overweight/obesity during catch-up growth. In this study, the overweight and non-overweight subjects were born with similar weight SDS and height SDS. However, the growth pattern of the overweight group was different from that of the non-overweight group after three months. The overweight group had a smaller length/height SDS but a higher rate of the change in weight SDS during catch-up growth, which accounted for the difference in BMI at the age of two years. One of the possible reasons for the big difference after three months might be feeding. SGA infants who were shorter at three months may be subsequently fed more if their parents attributed short stature to under nutrition. The results could be more practical if the parents of SGA subjects monitor the weight SDS and height SDS of their children, especially at the age of three months. Those who had lower SDS in height should pay more attention to weight control in the upcoming months. Therefore, parameters at three months of age might be competitive predictors for overweight/obesity in childhood as well as in adulthood, enabling early measures to prevent overweight/obesity^31^. To the best of our knowledge, this is the first study to report height at three months as the inflection point of overweight/obesity during catch-up growth in SGA infants.

This study had several limitations. First, this was an observational study and no intervention was allowed. Therefore, we used multivariate logistic regression to minimize the background noise. Second, more information about the subjects’ parents should be collected, such as height, which can help to identify the subjects who were constitutionally small at birth and the stratification for further investigation. Third, longer follow-up duration could facilitate a better understanding of overweight/obesity during catch-up growth.

In summary, this is the first study to propose that height at three months can be a predictor for overweight/obesity during the first two years. SGA subjects with lower length/height at three months had a higher prevalence of overweight at the age of two years. Therefore, it is necessary to monitor the growth pattern of SGA subjects with lower height SDS at three months in case of overweight/obesity and metabolic syndrome.
